# Editorial: Understanding public discourse for digital mental health promotion

**DOI:** 10.3389/fdgth.2023.1208116

**Published:** 2023-05-16

**Authors:** Ang Li, Xiaoqian Liu, Xingyun Liu

**Affiliations:** ^1^ Department of Psychology, Beijing Forestry University, Beijing, China; ^2^ Institute of Psychology, Chinese Academy of Sciences, Beijing, China; ^3^ School of Psychology, Central China Normal University, Wuhan, China

**Keywords:** digital mental health, technology innovation, health technology implementation, barrier, solution

**Editorial on the Research Topic** Understanding public discourse for digital mental health promotion

As mental health plays a pivotal role in achieving global sustainable development goals, mental health care is under more pressure than ever before. Unfortunately, the scarcity of mental health clinicians is expected to persist globally. However, emerging digital technologies are rapidly revolutionizing healthcare. In recent years, there has been a significant increase in both academic and clinical interest in the use of digital technologies to advance mental health in previously unimaginable ways. Indeed, when implemented correctly, digital technologies provide us with fresh insights into the experiences of people living with mental illnesses, enable clinicians to make more informed decisions based on accurate information, and facilitate more personalized delivery of interventions and resources.

Nevertheless, the cautionary tale of IBM Watson illustrates the dangers of technology that prioritizes marketing over results. Despite the potential of digital health technologies to improve the access and quality of mental health care, there is little evidence that such innovations can be successfully implemented in real clinical practice as substantial uncertainties remain.

This Research Topic aimed to advance knowledge about barriers, facilitators, and solutions for implementing digital mental health innovations in clinical settings, and it has received five submissions. The five insightful articles in this Research Topic, written by 16 influential researchers from the Netherlands and China, along with the excellent work of our experienced editors and peer reviewers from Canada, China, Germany, Iraq, and the United Kingdom, provide opportunities for new discoveries in the discipline in three specific areas:

• Strategies to overcome barriers in digital mental health
• Innovations to advance better digital mental health practice
• Mechanisms for mental health promotion on the Internet

In the first article, authors from the Netherlands, Sofia Bastoni et al. elaborated on the *Implementation of eMental health technologies for informal caregivers: A multiple case study*. They considered informal caregivers as an example and discussed effective ways of dealing with the challenges of digital mental health implementation. It has been suggested that “constructing a business model” and “discussing tool maintenance and long-term hosting in advance” could be possible ways of overcoming barriers in digital mental health.

In the second article, authors from China, Sihua Lyu et al. authored *Detecting depression of Chinese microblog users* via *text analysis: Combining Linguistic Inquiry Word Count (LIWC) with culture and suicide related lexicons*. By using social media data, they proposed training a computational model for predicting depression using a wider range of linguistic features and demonstrated the importance of incorporating culture-related and suicide-related linguistic features into depression model training.

In the third article, authors from China, Mengyao Song and Nan Zhao explained their work on *Predicting life satisfaction based on the emotion words in self-statement texts.* They attempted to establish a computational model for predicting life satisfaction by analyzing emotion-related word frequency in freestyle writing and improved the generalizability of the prediction model.

In the fourth article, authors from China, Qun Ye et al. investigated the effects of an online mindfulness-enhanced course designed to reduce stress in teachers. Their empirical study, *Validation of an online mindfulness-enhanced course for stress reduction in teachers*, supported the reliability and validity of developing a brief online course to promote mental health.

Finally, authors from China, Jun Zhan et al. wrote *The relationship between cyberbullying victimization and cyberbullying perpetration: The role of social responsibility*, highlighting the role of social responsibility in reducing the harmful effects of cyberbullying, a mental phenomenon in the information era.

The articles in this Research Topic provide valuable insights into ways to address the challenges of implementing digital mental health and introduce the potential of a diverse range of digital technologies and empirical findings to aid in promoting mental health. Despite the fact that there is still a long way to go before the full potential of digital mental health can be realized, we would like to express our appreciation to the authors for their contributions to this Research Topic, and we look forward to continuing the dialogue and collaboration on this important topic.

